# Supplementary material for: From sequence to enzyme mechanism using multi-label machine learning
Source: BMC Bioinformatics. 2014 May 19;15:150. doi: 10.1186/1471-2105-15-150 (PMC4229970; doi:10.1186/1471-2105-15-150)
Supplement: Additional file 2 — Java code of ml2db. Additional file ml2db_code.tar.gz contains the Java source code to run the multi-label machine learning experiments and save the results to database. The code’s Javadoc is included. [file 1471-2105-15-150-S2.zip › additional file 2/ml2db/ml2db/doc/main/ml2db/mulan/predict/QuickPredictor.html]

QuickPredictor


JavaScript is disabled on your browser.


- Overview
- Package
- Class
- Use
- Tree
- Deprecated
- Index
- Help

- Prev Class
- Next Class

- Frames
- No Frames

- All Classes

- Summary:
- Nested |
- Field |
- Constr |
- Method

- Detail:
- Field |
- Constr |
- Method


main.ml2db.mulan.predict

## Class QuickPredictor

- java.lang.Object
- - main.ml2db.mulan.predict.QuickPredictor

- ---

    

  ```
  public class QuickPredictor
  extends java.lang.Object
  ```

  Input: 1. a data file path, 2. a serialised pre-trained Mulan classifier, 3.
  an output file path (to write the predictions to). No spaces are allowed in
  the file names.
  Data file format: a tab separated file with: instance\_identifier,
  attribute\_identifier (optional third column: attribute\_value). If only the
  first two columns are present, the attribute value will be treated as sparse
  binary: if the attribute is associated with an instance, the attribute value
  will be 1. If the attribute is not associated with the instance, the
  attribute value will be treated as 0 and thus absent from the sparse instance
  (to save memory and disk space). If a third column is present in the data
  file, the (numerical) value in the third column will be treated as the
  attribute value. Attributes present in the pre-trained classifier but not
  associated to an instance the data file will be treated as missing ("?" in
  Weka ARFF format) for that instance.
  Output: a tab separated file with: instance\_identifier, class\_label,
  probability\_of\_prediction
  Beware, the experiment will not be logged in the database! The preferred mode
  of use of ml2db is to fill a properties file containing the sql queries to
  get the data from database. The filled .props file can then be used as input
  for the MulanArff class to generate and save to database an ARFF file. The
  resulting ARFF identifier can then be used for cross-validation, train-test
  experiments, prediction etc. This guarantees full database logging of data,
  experiments, predictions etc. for analysis and reuse.

  Version:
  :   2 Aug 2013

  Author:
  :   Luna De Ferrari ldf@st-andrews.ac.uk

- - ### Constructor Summary

    Constructors

    Constructor and Description

    `QuickPredictor()`
  - ### Method Summary

    Methods

    | Modifier and Type | Method and Description |
    | --- | --- |
    | `static void` | `main(java.lang.String[] args)` To run a prediction experiment, please provide: Input: 1. |

    - ### Methods inherited from class java.lang.Object

      `equals, getClass, hashCode, notify, notifyAll, toString, wait, wait, wait`

- - ### Constructor Detail


    - #### QuickPredictor

      ```
      public QuickPredictor()
      ```
  - ### Method Detail


    - #### main

      ```
      public static void main(java.lang.String[] args)
      ```

      To run a prediction experiment, please provide:
      Input: 1. a data file path, 2. a serialised pre-trained Mulan classifier,
      3. an output file path (to write the predictions to). No spaces are
      allowed in the file names.
      Data file format: a tab separated file with: instance\_identifier,
      attribute\_identifier (optional third column: attribute\_value). If only
      the first two columns are present, the attribute value will be treated as
      sparse binary: if the attribute is associated with an instance, the
      attribute value will be 1. If the attribute is not associated with the
      instance, the attribute value will be treated as 0 and thus absent from
      the sparse instance (to save memory and disk space). If a third column is
      present in the data file, the (numerical) value in the third column will
      be treated as the attribute value. Attributes present in the pre-trained
      classifier but not associated to an instance the data file will be
      treated as missing ("?" in Weka ARFF format) for that instance.
      Output: a tab separated file with: instance\_identifier, class\_label,
      probability\_of\_prediction

      Parameters:
      :   `args` -


- Overview
- Package
- Class
- Use
- Tree
- Deprecated
- Index
- Help

- Prev Class
- Next Class

- Frames
- No Frames

- All Classes

- Summary:
- Nested |
- Field |
- Constr |
- Method

- Detail:
- Field |
- Constr |
- Method
